# Supplementary material for: Mapping brain function underlying naturalistic motor observation and imitation using high-density diffuse optical tomography
Source: Imaging Neurosci (Camb). 2025 Sep 19;3:IMAG.a.153. doi: 10.1162/IMAG.a.153 (PMC12451301; doi:10.1162/IMAG.a.153)
Supplement: Supplementary Material [file IMAG.a.153_supp.pdf]

Supplementary Materials

Regarding handedness, the ratio of right- to left-handed individuals in our overall sample was 10.3% (10/97), closely aligning with the general population's handedness distribution of 10.6% (Papadatou-Pastou et al., 2020). Moreover, there was no significant difference between the NAI and ASD groups with respect to age (ASD $n = 19$, NAI $n = 81$, $t = 1.37$, $p = 0.17$), sex (ASD $n = 19$, NAI $n = 81$, $p = 0.12$), or handedness (ASD $n = 19$, NAI $n = 78$, $p = 0.41$). Furthermore, significant differences in GM were found across five race groups (F = 10.01, $p < 0.001$, white $n = 75$, Asian $n = 17$, more than one race $n = 4$, black $n = 2$, and unknown $n = 2$), with post-hoc t-tests revealing a significant difference between Asian and white groups (Cohen's D = -1.21, $p < 0.001$), while a significant difference in median SNR was found across races (F = 2.86, $p = 0.04$). However, no significant differences were observed in either median SNR (F = 0.68, $p = 0.51$) or GM (F = 0.61, $p = 0.55$) across ethnic groups (not Hispanic/Latino $n = 93$, Hispanic/Latino $n = 5$, unknown $n = 2$), and although there was an overall difference in median SNR across racial groups, no pairwise comparisons were significant in the post-hoc analysis.

Supplementary Figure 1. Behavioral Assessments of motor performance. A Participant performing imitation of movements. **B** Three examples of extracted skeleton of moving participant for CAMI analyses. **C** Comparison of imitation performance with a gold-standard skeleton position during the movements

Supplementary Figure 2: Brain regions exhibiting significant responses during motor imitation but not motor observation. A. Brain regions with distinct significant activation (FDR corrected $p < 0.05$**)** during motor imitation that are not significantly active during motor observation. **B.** Temporal profiles of the HbO (oxygenated hemoglobin) hemodynamic response for each task, with shaded areas representing the standard error of the mean across participants.

Supplementary Figure 3: Brain regions exhibiting significant responses during motor observation but not motor imitation. A. Brain regions with distinct significant activation (FDR corrected $p < 0.05$**)** during motor observation task that are not significantly active during motor imitation. **B.** Temporal profiles of the HbO (oxygenated hemoglobin) hemodynamic response for each task, with shaded areas representing the standard error of the mean across participants. Time courses include a 5-second pre-stimulus baseline period (–5 to 0 s), with stimulus onset at 0 s.

Supplementary Figure 4: Neural correlates of observation and imitation within the mirror neuron system. A. Parcel-based analysis of the MNS within the field of view, highlighting regions activated during both motor observation and imitation. **B.** FDR-corrected t-map showing the statistically significant regions within the MNS, with false discovery rate correction applied to control for multiple comparisons.

Supplementary Figure 5: Functional network involvement during motor observation and imitation. A Task specific significant brain parcels under three conditions (OBS, IM, and their contrast). **B** Functional networks involved during the OBS task. **C** Functional networks involved during the IM task. **D** Functional networks more active during the OBS task than the IM task. **E** Functional networks more active during the IM task than the OBS task.

Supplementary Figure 6: Neural correlates of OBS and IM within dorsal attention network and ventral attention network. A. FDR-corrected t-map for motor observation (N = 77), motor imitation (N = 63), and their contrast (red indicates brain regions where OBS > IM, highlighting statistically significant regions within DAN with false discovery rate correction applied to account for multiple comparisons. **B.** FDR-corrected t-map for motor observation (N = 77), motor imitation (N = 63), and their contrast (red indicates brain regions where OBS > IM, highlighting statistically significant regions within VAN with false discovery rate correction applied to account for multiple comparisons.

Supplementary Figure 7: Group-level activation maps in NAI-only participants. A Unthresholded *t*-maps for motor observation (N = 64), motor imitation (N = 50), and imitation vs. observation contrast. **B** Parcels with significant activation after FDR correction ($p < 0.05$). Spatial activation patterns are consistent with those observed in the full sample, supporting the generalizability of the main findings.

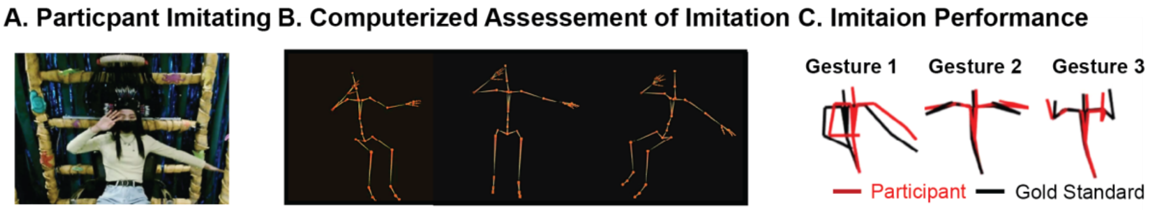

Supplementary Figure 1. Behavioral Assessments of motor performance. A Participant performing imitation of movements. **B** Three examples of extracted skeleton of moving participant for CAMI analyses. **C** Comparison of imitation performance with a gold-standard skeleton position during the movements.

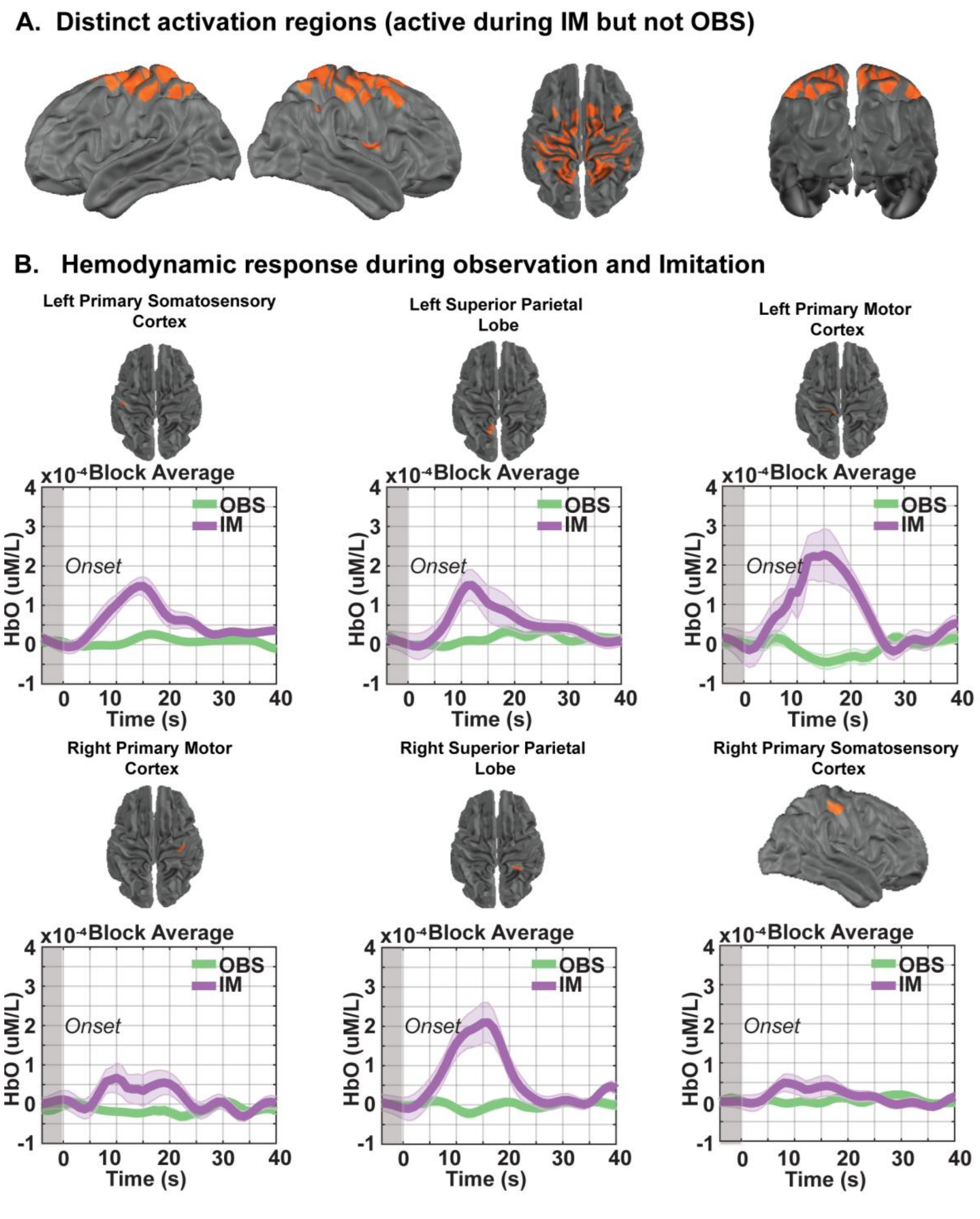

Supplementary Figure 2: Brain regions exhibiting significant responses during motor imitation but not motor observation. A. Brain regions with distinct significant activation (FDR corrected $p < 0.05$**)** during motor imitation that are not significantly active during motor observation. **B.** Temporal profiles of the HbO (oxygenated hemoglobin) hemodynamic response for each task, with shaded areas representing the standard error of the mean across participants. Time courses include a 5-second pre-stimulus baseline period (–5 to 0 s), with stimulus onset at 0 s.

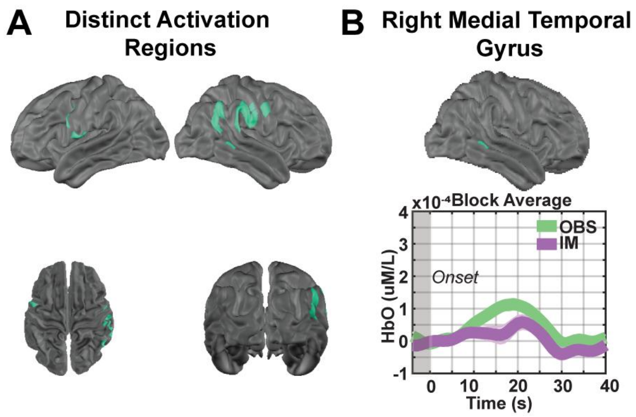

Supplementary Figure 3: Brain regions exhibiting significant responses during motor observation but not motor imitation. **A.** Brain regions with distinct significant activation (FDR corrected $p < 0.05$**)** during motor observation task that are not significantly active during motor imitation. **B.** Temporal profiles of the HbO (oxygenated hemoglobin) hemodynamic response for each task, with shaded areas representing the standard error of the mean across participants. Time courses include a 5-second pre-stimulus baseline period (–5 to 0 s), with stimulus onset at 0 s.

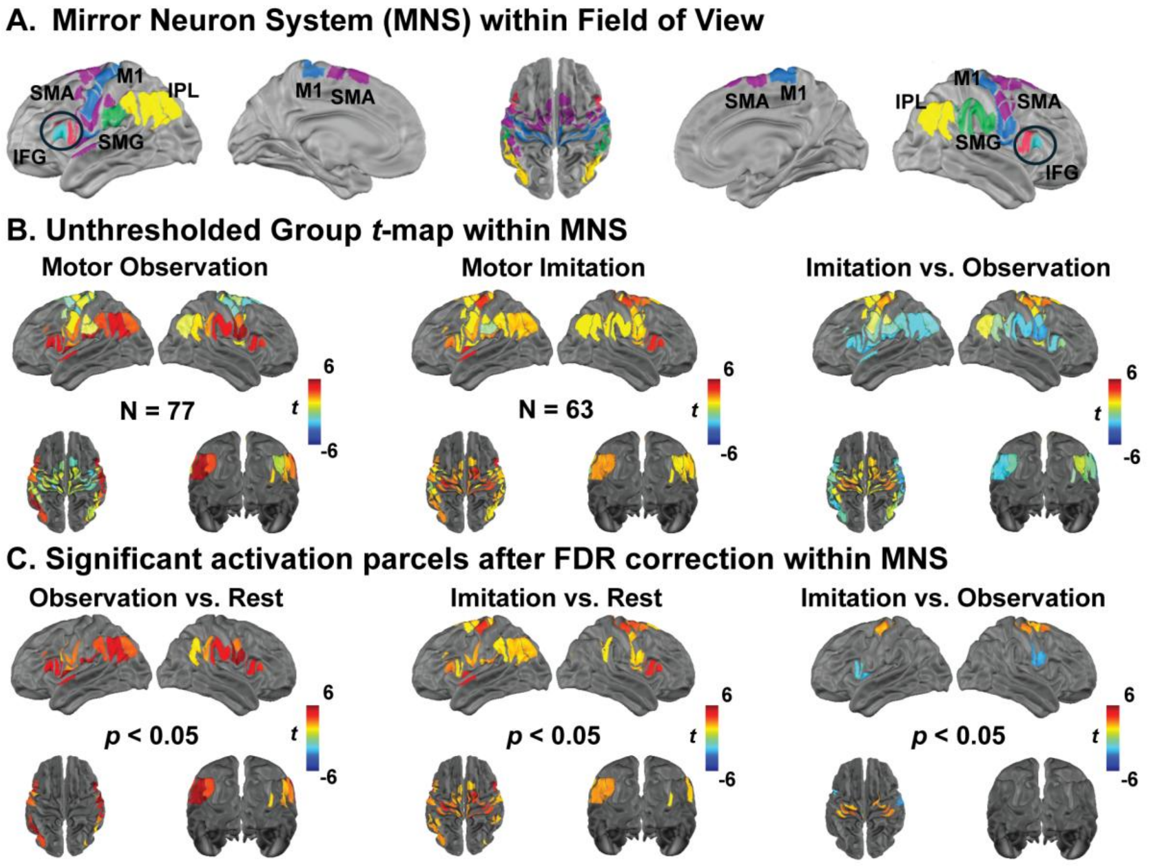

Supplementary Figure 4: Neural correlates of observation and imitation within the mirror neuron system. A. Parcel-based analysis of the MNS within the field of view, highlighting regions activated during both motor observation and imitation. **B.** FDR-corrected t-map showing the statistically significant regions within the MNS, with false discovery rate correction applied to control for multiple comparisons.

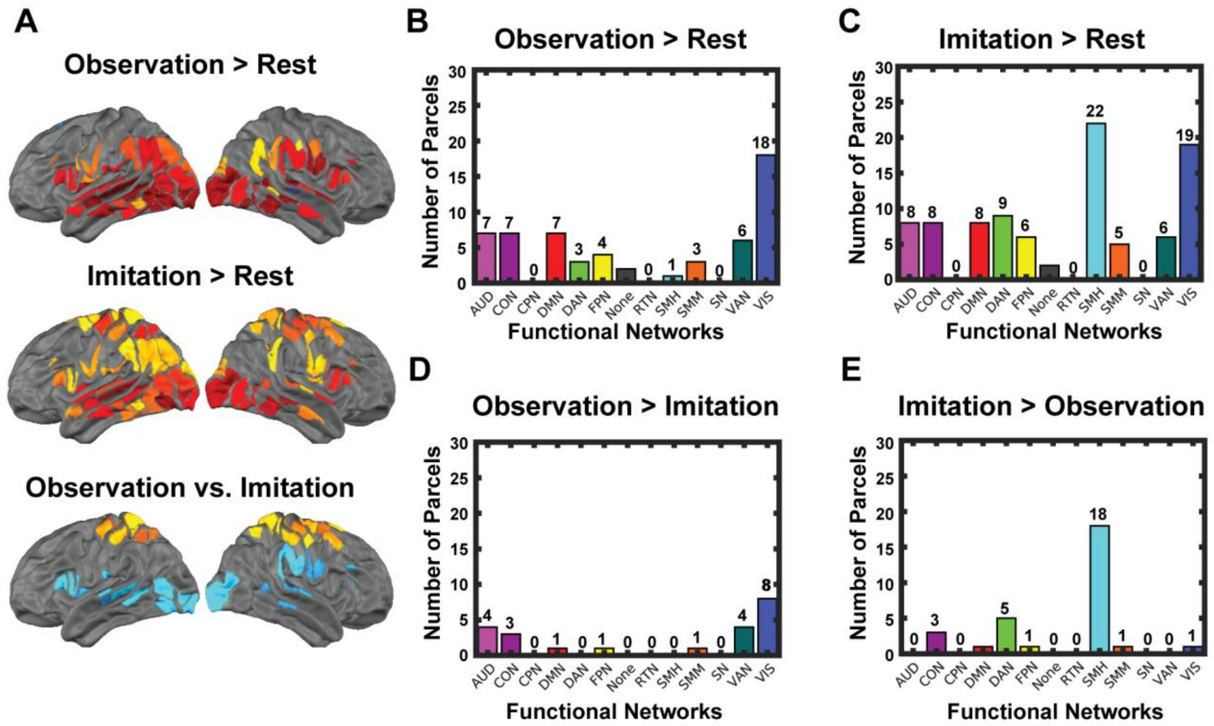

Supplementary Figure 5: Functional network involvement during motor observation and imitation. A Task specific significant brain parcels under three conditions (OBS, IM, and their contrast). **B** Functional networks involved during the OBS task. **C** Functional networks involved during the IM task. **D** Functional networks more active during the OBS task than the IM task. **E** Functional networks more active during the IM task than the OBS task.

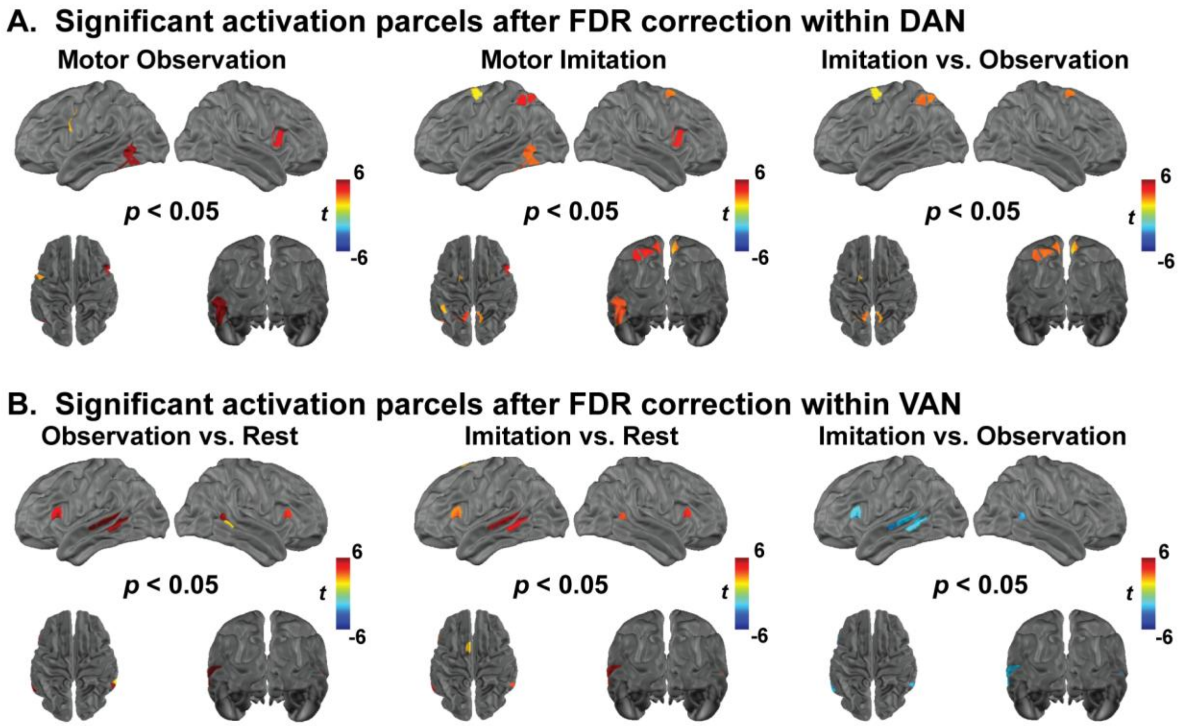

Supplementary Figure 6: Neural correlates of OBS and IM within dorsal attention network and ventral attention network. A. FDR-corrected t-map for motor observation (N = 77), motor imitation (N = 63), and their contrast (red indicates brain regions where OBS > IM, highlighting statistically significant regions within DAN with false discovery rate correction applied to account for multiple comparisons. **B.** FDR-corrected t-map for motor observation (N = 77), motor imitation (N = 63), and their contrast (red indicates brain regions where OBS > IM, highlighting statistically significant regions within VAN with false discovery rate correction applied to account for multiple comparisons.

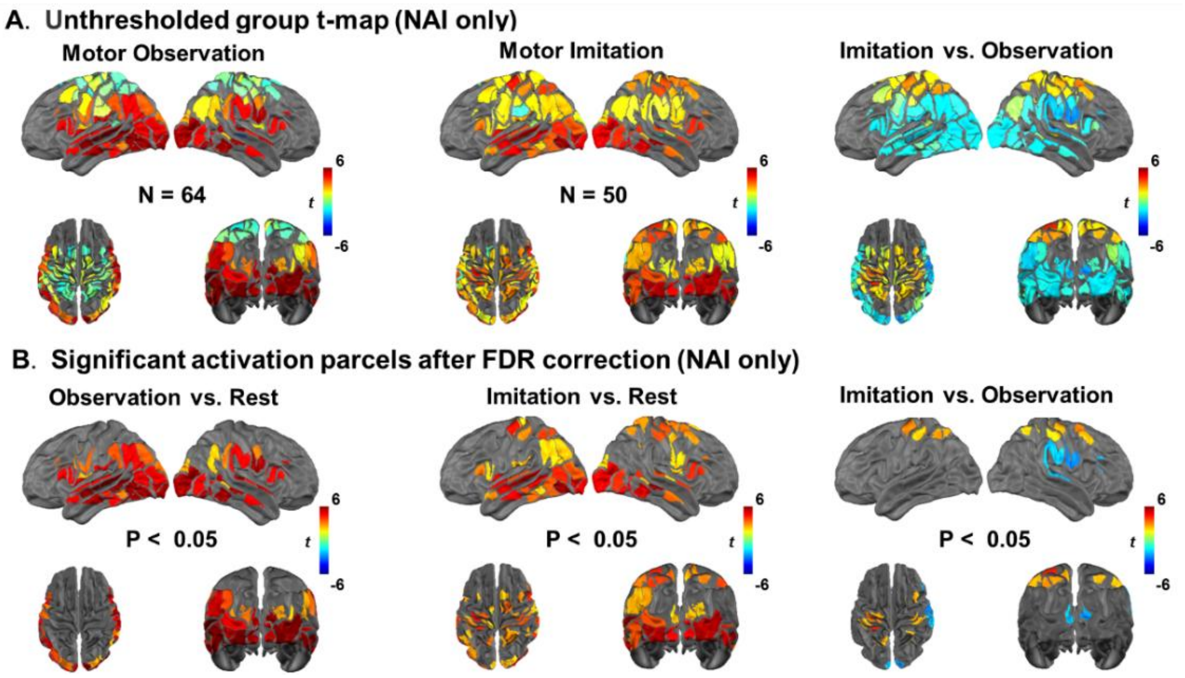

Supplementary Figure 7. Group-level activation maps in NAI-only participants. (**A**) Unthresholded *t*-maps for motor observation (N = 64), motor imitation (N = 50), and imitation vs. observation contrast. (**B**) Parcels with significant activation after FDR correction ($p < 0.05$). Spatial activation patterns are consistent with those observed in the full sample, supporting the generalizability of the main findings.

Supplementary Table 1: Summary of participant inclusion and retention across data analyses.

Analysis group	Inclusion/exclusion Criteria	Total (n)	ASD (n)	NAI (n)
Initial enrollment	Full cohort	100	17	83
Valid CAMI data	11 excluded: damaged video (6), no video (2), CAMI preprocessing failure (3)	89	17	72
Valid SRS-2 scores	9 participants missing SRS-2	91	13	78
High data quality: Observation	GM > 80%, SNR > 1	78	13	65
High data quality: Imitation	GM > 80%, SNR > 1	64	13	51
Final sample: Observation	After removing stimulus error case	77	13	64
Final sample: Imitation	After removing stimulus error case	63	13	50
Brain–behavior analysis (Observation + SRS-2)	High data quality and completed SRS-2	71	13	58
Brain–behavior analysis (Imitation + SRS-2)	High data quality from the first DOT run and completed SRS-2	55	12	43
Brain–behavior analysis (Imitation + CAMI)	High data quality from the first DOT run and CAMI score existed	48	11	37

Supplementary Table 2: Summary of Statistical Results for Brain–Behavior Associations with SRS-2 Scores

	Pearson Correlation		Multiple regression model (SRS, Age, sex, and handedness)						
Brain Region	*R*	*p-value*	*F*	*R^2 / adj. R^2*	*p-value*	*β(SRS-2)*	*t (SRS-2)*	*p (SRS-2)*	*Covariates*
Right SPL*	0.46	3.40E-05	5.18	0.24 / 0.19	<0.001	0.43	3.94	<0.001	None
Right IPL	0.24	0.045	5.18	0.24 / 0.19	<0.001	0.433	3.94	<0.001	Handedness
Left IPL	0.24	0.045	1.06	0.06 / 0.003	0.39	0.23	1.6	0.11	None
Left SMA	0.25	0.03	2.315	0.12 / 0.07	0.07	0.27	2.1	0.04	None
Right SMA	0.28	0.01	1.72	0.09 / 0.04	0.16	0.29	2.4	0.02	None
Left Occipital	-0.31	0.009	2.09	0.11 / 0.06	0.09	-0.32	2.66	0.01	Sex
Right Occipital	-0.24	0.04	6.42	0.28 / 0.24	<0.001	-0.4	3.75	<0.001	None
Left Subcentral Gyrus	-0.3	0.03	3.33	0.21 / 0.15	0.02	-0.38	2.857	0.006	Sex

* Passed the FDR correction (p = 0.007)

Supplementary Table 3: Summary of Statistical Results for Brain–Behavior Associations with CAMI scores

	Pearson Correlation		Multiple regression model (CAMI, Age, sex, and handedness)						
Brain Region	*R*	*p-value*	*F*	*R^2 / adj. R^2*	*p-value*	*β(SRS-2)*	*t (SRS-2)*	*p (SRS-2)*	*Covariates*
Left FEF	0.43	0.002	4.96	0.32 / 0.26	0.002	0.439	3.3	0.002	Age
Right IPL	0.31	0.03	2.83	0.21 / 0.14	0.04	0.37	2.58	0.01	Handedness
Right M1	-0.29	0.04	2.28	0.18 / 0.10	0.13	-0.31	2.1	0.04	None
Left FFG	0.34	0.02	1.9	0.21 / 0.14	0.07	-0.31	2.13	0.04	None
